# A preliminary study on the distribution patterns of endemic species of Fulgoromorpha (Hemiptera, Auchenorrhyncha) in Iran

**DOI:** 10.3897/zookeys.319.4159

**Published:** 2013-07-30

**Authors:** Fariba Mozaffarian

**Affiliations:** 1 Insect Taxonomy Research Department, Iranian Research Institute of Plant Protection, Tehran, 19395, P.O. Box 1454, Iran

**Keywords:** Zoogeography, Iran, Fulgoromorpha

## Abstract

Iran is known as the most complex and varied country in southwest Asia, in terms of geography, vegetation, climate and consequently biological diversity. The rather high number of recorded endemic species of Fulgoromorpha in Iran indicates a high potential for speciation in some areas.

In this study, in order to identify the endemic zones for Fulgoromorpha of Iran, three main biogeographic regions of the country were divided into 13 primary zones, mainly according to the distribution of published and unpublished locality records of endemic species. Using Venn diagrams and cluster analyses on the primary zones, 6 final endemic zones were recognized: Caspian zone, southern slopes of Alborz, Zagros Mountains, Kerman Mountains, Khorasan Mountains, and Baluchestan and Persian Gulf coasts. Then a similarity map was produced for endemic zones using a Multidimensional analysis (Alscal) and the differences between the positions of the same zones in the similarity and geographic maps were discussed.

## Introduction

Iran is located in southwest Asia between the latitudes of 25°30' and 40° north and the longitudes of 44° and 63°30' east and has a surface area of 1,648,195 square kilometers. It is limited by central Asia, Caspian Sea, Caucasus Mountains and Aras River to the north, Anatolian Plateau and Mesopotamian region to the west, Persian Gulf and Oman Sea which is connected to the Indian Ocean to the south and Afghanistan and Pakistan to the east. The major part of the land of Iran is covered by the Plateau of Iran, a triangle between the Alborz Mountain Range in the north, the Zagros Mountains in the west and the Sulaiman Mountain Range in Pakistan and Afghanistan.

According to Manuel Berberian (1981), the continental crust of Iran has been originally developed by some complex movements which made the plate of the Alborz and central Iran and then the Zagros and Arabian plate detach from the Gondwana supercontinent in the southern hemisphere, drift northwards and attach to Eurasia from Permian to late Miocene. Consequently some parts of the old Tethys have been recognized in some parts of the country (Ghorbani 2002).

In terms of biodiversity richness, Iran is considered as an extremely complex area and wide ranges in the extremes of altitude (below sea-level in shores of the Caspian sea to 5,770 m of the Damavan Mt.), climate (humid and nearly jungle-like forests in the north to arid places in Dasht-e Lut with less than 100 m annual rainfall) and temperature (from -35°C in the north west to 70°C in the deserts of Dasht-e Lut) (Hedge and Wendelbo 1978, Frey and Probst 1986, Zohary 1963). The high biodiversity of the Iranian fauna is also the result of its location and the influences of four ecozones from north (Palaearctic and old periodical connections with the Nearctic by Bering Strait), south (Afrotropical from the Arabian Peninsula) and southeast (Oriental) (Madjnoonian et al. 2005).

**Figure 1.**
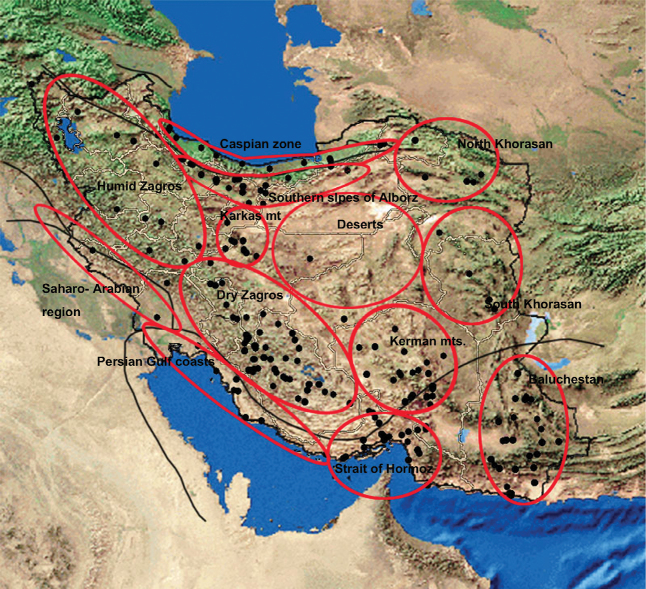
Distribution of endemic Fulgoromorpha of Iran, Phytogeographical regions (Black lines) (after Zohary 1973, altered by Frey and Probst 1986 and Primary divisions (Red lines) suggested for the distribution pattern of endemic planthoppers of Iran.

Biogeographic studies in Iran have been carried out mainly on plants (Zohary 1963, 1973, Frey and Probst 1886, Hedge and Wendelbo 1978, Hangay and Szekely 2005). Those studies recognized three main biogeographic regions in Iran which are usually applied as useful framework and references in analyses: Hyrcanian, Irano-Turanian and Nubo-Sindian and also a small area in the west of Iran which is touched by the Saharo-Arabian region according to Zohary (1973) (Fig. 1).

The oldest zoogeographic division was performed by Zarudny (1911), an ornithologist who organized some trips to different parts of Iran during 1884-1904 and divided Iran into 9 zones: South Caspian Sea, Northwest, Khorasan, Kerman, Southwest, Sistan, Baluchestan, Karun plains and South coasts. In 1968, Anderson recognized 13 zones in his extensive study on endemic lizards of Iran: the central plateau, the Urmia basin, the Sistan basin, the Caspian region, the Khuzestan plain and Persian Gulf coast, Baluchestan and Makran coast, the Turkmen steppe, the Mogan steppe, the Zagros, the western foothills of the Zagros, the Kope Dag, the Alborz, the Islands of the Persian Gulf. Emeljanov (1974) considered Iran in the Sethian Desert Region in his Palaearctic biogeographic divisions and showed 2 subregions inside Iran: Sahara-Arabian (Makran mixed and Sind plane) and Irano-Turanian (Middlear Eastern mixed (Armenian and Zagrossan), Hyrcanian Mountain, Iranian Mixed and Khorasan Mountain). Dlabola (1981) classified different habitats in Iran and described the origin of studied Auchenorrhyncha species in three groups: arboreal, eremian and oreal.

A total of 235 species of Fulgoromorpha have been recorded from Iran since 1902 (Mozaffarian and Wilson 2011), 117 of which from 13 families have never been recorded from other parts of the world and are restricted to the borders of Iran according to our knowledge. The high endemism (nearly 50%) in the recorded fauna of Iran shows a high potential of speciation inside Iran. The aim of this study is recognizing distribution patterns and endemic areas for endemic species of Fulgoromorpha in Iran.

## Materials and methods

A total of 473 locality records of 117 species of planthoppers endemic to Iran were gathered, 416 of which derived from publications, 48 were newly collected and identified species in the Hayk Mirzayans Insect Museum (HMIM), and 9 were studied in the Zoological Institute of the Russian Academy of Science (ZIN). Due to the lack of the exact locality data of 3 endemic species: *Phantia lactea* Rusiecka, 1902 (Flatidae), *Phantia putoni* Rusiecka, 1902 (Flatidae) and *Oliarus convergens* (Melichar, 1902) (Cixiidae), they were deleted from further analyses. Then the distribution maps were prepared using Arc Map 9.3. and the country was divided into 13 primary zones according to the distribution of the endemic planthoppers, topography, climate condition and published zoogeographic zones (Fig. 1). Venn diagrams and cluster analyses were performed using Gliffy venn diagram software and NTSYS (2.02g) (Rohlf 1998) to find the relationship between those primary zones and to identify the final zones. The input data for cluster analyses were qualitative data: 1 and 0 for presence and absence of any species in any division, respectively. The similarity between final endemic zones was shown by a similarity map using a multidimensional scaling analysis (Alscal) by SPSS.

## Results and discussion

### Hyrcanian district

This is a small area in northern Iran, from the Caucasian-Euxino-Hyrcanian province of the Euro-Siberian region (Frey and Probst 1986). The district is limited to the Alborz Mountain and Caspian Sea, stretches from Talesh in Azarbaijan in the west to Gorgan in the east and characterized by predominantly deciduous forests.

**Figure 2.**
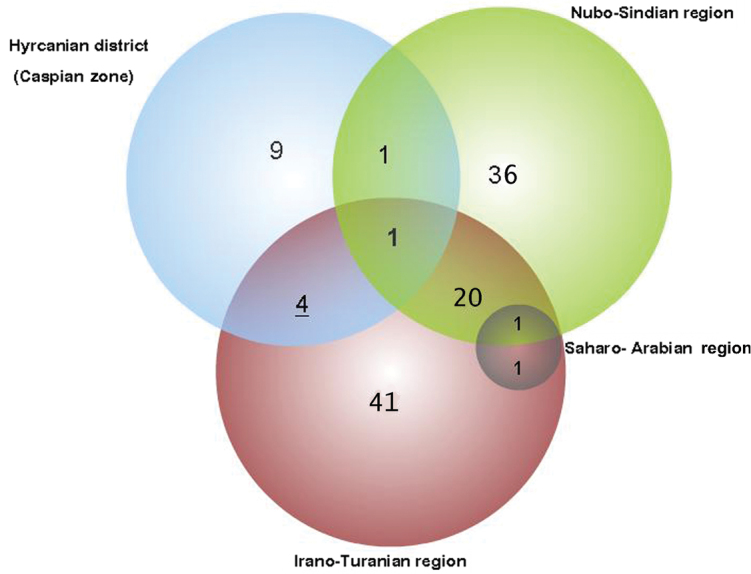
The number of endemic planthoppers of Iran in the main biogeographic areas.

The district is considered as one zone (Caspian zone) in this study. Fifteen endemic planthoppers of Iran have been recorded from this zone, 9 of which are endemic to the region (Table 1 and Fig. 2). The high and wall-like Alborz Mountain separates the south coast of the Caspian Sea from the Irano-Turanian region. This barrier not only prevents the fauna from migrating to south parts but also limits the high humidity of the Caspian Sea to the north and produces two very different climatic regions in the northern and southern slopes which can be considered a major factor for differentiating the fauna of north Iran from other parts of the country. Most of the Iranian endemic species common between Hyrcanian and other regions are limited to the Alborz Mt. and are distributed in areas very close to the borders of the Hyrcanian district. *Malenia masirica* Dlabola, 1986 (Derbidae) is the only endemic planthopper recorded in all three main regions. There are only a few records of the presence of this species in Iran which belong mostly to the southern Zagros, the Nubo-Sindian region, close to Irano-Turan, and one record of a female specimen from the north of Iran, all made by Dlabola (1986), and the specimens are deposited in Prague. *Morsina persica* Melichar, 1902 (Nogodinidae) is the only endemic planthopper common between the Caspian zone and the Nubo-Sindian in spite of the distance and lack of records in the Irano-Turanian. The species was originally described from south east of Iran by Melichar (1902) and there are also other published and unpublished records of this species from there, deposited in HMIM. However the only record of the presence of this species in the Hyrcanian district is made by Dlabola (1981) and he mentioned the species as an eremian faunal element which can be often found as oreal. According to this reference, the only specimen of this species from the north of Iran belongs to the museum of Plant Pests and Disease Research Institute, Tehran-Evin which is the former name used for HMIM, while it is not mentioned in the list of museum specimens (Mirzayans 1995) and is not currently existing in this museum either. However another specimen of this species is deposited in the HMIM museum collected from southeast of Iran, by the same collector and in the same date mentioned by Dlabola’s northern specimen. Therefore the presence of this species in the Hyrcanian district is doubtful and appears to be a mistake in recording the locality in Dlabola (1981).

**Table 1.** List of endemic species of Iran, indicating endemic species of suggested endemic zones (Extracted from Mozaffarian and Wilson 2011 and new data).

Endemic species	Caspian zone	Kerman Mountains	Zagros mountains	Khorasan Mountains	Southern Slopes of Alborz	Baluchestan and Persian Gulf coasts
**Family: Caliscelidae**						
*Adenissus baluchestanicus* Dlabola, 1980						*
*Adenissus isinus* Dlabola, 1980						*
*Adenissus zabolicus* Dlabola, 1980				*		
*Adenissus zahedanicus* Dlabola, 1980						*
*Aphelonema brunneolutea* Dlabola 1994					*	
*Perissana dlabolai* Gnezdilov & Wilson, 2006						
*Reinhardema pasagarda* (Dlabola, 1982)			*			
**Family: Cixiidae**						
*Duilius v-atrum* (Dlabola, 1985)						
*Cixius persicus* Distant, 1907				*		
*Myndus genocolus* Dlabola, 1985						*
*Myndus sarbazus* Dlabola, 1989						*
*Anoculiarus ornatus* Dlabola, 1985		*				
*Eumecurus apunctatus* Dlabola, 1985						
*Eumecurus baluchestanicus* Dlabola, 1985			*			
*Eumecurus octopus* Dlabola, 1985						*
*Eumecurus superstylus* Dlabola, 1985		*				
*Eumecurus transpunctatus* Dlabola, 1985						*
*Eumecurus vilbastei* Dlabola, 1985						*
*Hyalesthes restultus* Dlabola, 1994			*			
*Hyalesthes zabolicus* Dlabola, 1985						
*Oliarus convergens* Melichar, 1902						
*Pentastira bahtiaricus* (Dlabola, 1981)			*			
*Pentastira superspicata* Dlabola, 1985			*			
*Pentastira shul* (Dlabola, 1985)			*			
*Reptalus eremicus* Dlabola, 1985						
*Reptalus reductus* Dlabola, 1994		*				
*Reptalus ziaran* Dlabola, 1985					*	
**Family: Delphacidae**						
*Tropidocephala prasina* Melichar, 1902						*
*Muirodelphax amol* Dlabola, 1981	*					
*Pseudaraeopus curtulus* Dlabola, 1960						*
*Pseudaraeopus iranicus* Dlabola, 1960						*
*Ribautodelphax hyrcanus* Dlabola, 1981	*					
**Family: Derbidae**						
*Malenia isinica* Dlabola, 1986						*
*Malenia masirica* Dlabola, 1986						
*Proutista jezeki* Dlabola, 1981						*
**Family: Dictyopharidae**						
*Callodictya kazeruna* (Dlabola, 1986)			*			
*Dictyophara albata* Dlabola & Heller, 1962						
*Dictyophara exoptata* Dlabola & Heller, 1962						
*Dictyophara pazukii* (Dlabola, 1984)			*			
*Dictyophara hoberlandti* Dlabola, 1974						
*Kumlika mandrita* Emeljanov, 1997				*		
*Nymphorgerius convergens* Emeljanov, 1972						
*Nymphorgerius emeljanovi* Dlabola, 1979					*	
*Nymphorgerius mullah* Dlabola, 1979					*	
*Nymphorgerius rostratus* Emeljanov, 2009						
**Family: Flatidae**						
*Bahuflata punctata* Dlabola, 1979						*
*Derisa atratula* Melichar, 1902						
*Mesophantia kanganica* Dlabola, 1983						
*Mesophantia pallens* Melichar, 1902						
*Mesophantia sabzevaranica* Dlabola, 1983						
*Mesophantia tisina* Dlabola, 1983						
*Persepolia jasmuriana* Dlabola, 1982						*
*Persepolia secunda* Dlabola, 1981						*
*Persepolia servadeina* Dlabola, 1982						
*Phantia borazianica* Dlabola, 1989						*
*Phantia crucispina* Dlabola, 1989						*
*Phantia denasuta* Dlabola, 1989						*
*Phantia finita* Dlabola, 1989			*			
*Phantia helleri* Linnavuori, 1962						
*Phantia lactea* Rusiecka, 1902						
*Phantia ovatospina* Dlabola, 1989				*		
*Phantia picea* Dlabola, 1989						*
*Phantia putoni* Rusiecka, 1902						
*Tisia esfandiarii* Dlabola, 1981						*
**Family: Issidae**						
*Cavatorium ardakanum* Dlabola, 1980			*			
*Cavatorium bispinatum* Dlabola, 1980			*			
*Cavatorium quadrispinatum* Dlabola, 1980			*			
*Cavatorium sarbaz* Dlabola, 1980						*
*Eusarima iranica* Gnezdilov & Mozaffarian 2011					*	
*Inflatodus astyages* Dlabola, 1982					*	
*Inflatodus kyaxares* Dlabola, 1982					*	
*Inflatodus persicus* (Dlabola, 1981)					*	
*Inflatodus viridans* (Dlabola, 1974)					*	
*Iranodus amygdalinus* Dlabola, 1980						
*Iranodus dumetorus* (Dlabola, 1981)			*			
*Iranodus khatunus* (Dlabola, 1981)						
*Iranodus nishabur* Dlabola, 1982				*		
*Iranodus repandus* (Dlabola, 1981)			*			
*Iranodus transversalis* Dlabola, 1980		*				
*Mycterodus astragalicus* Dlabola, 1974			*			
*Mycterodus demavendinus* Dlabola, 1981					*	
*Mycterodus elbursicus* (Logvinenko, 1974)	*					
*Mycterodus fagetophilus* Dlabola, 1980	*					
*Mycterodus guilanicus* Dlabola, 1981	*					
*Mycterodus hezarmeshedi* Dlabola, 1980		*				
*Mycterodus inassuetus* Dlabola, 1981	*					
*Mycterodus kandavanicus* Dlabola, 1980						
*Mycterodus krameri* Dlabola, 1974						
*Mycterodus lanceatus* Dlabola 1997	*					
*Mycterodus peterseni* Dlabola, 1980	*					
*Mycterodus sexpunctatus* Dlabola, 1980						
*Mycterodus shahrudicus* Dlabola, 1980					*	
*Pentissus bamicus* Dlabola, 1980						
*Phasmena adyoungi* Dlabola, 1982						*
*Phasmena telifera* Melichar, 1902						*
*Quadriva aurita* (Dlabola, 1982)			*			
*Quadriva dehbakrina* (Dlabola, 1980)		*				
*Quadriva lassa* (Dlabola, 1981)			*			
*Quadriva proxima* (Dlabola, 1980)		*				
*Quadriva sabzevarana* (Dlabola, 1980)		*				
*Quadriva taftanica* (Dlabola, 1980)						*
*Quadriva tangesarhena* (Dlabola, 1980)						
*Scorlupaster emersum* (Dlabola, 1981)	*					
**Family: Kinnaridae**						
*Perloma boroumandi* (Dlabola, 1981)						*
*Perloma satrapa* (Dlabola, 1981)						
*Perloma zarudnyi* (Emeljanov, 1984)						*
**Family: Meenoplidae**						
*Anigrus farsicus* Dlabola, 1986						*
**Family: Nogodinidae**						
*Hadjia nerii* Dlabola, 1981						*
*Hadjia quadrifasciata* Dlabola, 1981						*
*Iranissus ephedrinus* Dlabola, 1980						
*Morsina persica* Melichar, 1902						
*Philbyella glarea* Dlabola & Heller, 1962						
**Family: Ricaniidae**						
*Ricania soraya* Dlabola, 1983			*			
**Family: Tettigometridae**						
*Tettigometra demavenda* Dlabola, 1981					*	
**Family: Tropiduchidae**						
*Kazerunia leguaniforma* Dlabola, 1977						*
*Kazerunia ochreata* Dlabola, 1974			*			
*Kazerunia undulata* Dlabola, 1977						*

Historically, the north of Iran was occupied by the old Tethys 55-20 mya (Ghorbani, 2002) so the occupation should have occurred after the area got dry. During the Pliocene ice age (100,000 years ago) the region was used as a refugee, and species spread northwards after the ice age, which may be a reason for rather small numbers of endemic species in this region. Hedge and Wendelbo (1978) also indicated rather small numbers of endemic Phanerogamic plants in this area. However the area has a considerably different climate and fauna from other parts of Iran and has been considered as a separate zoogeographic division both by Zarudny (1911), Anderson (1968) and Emeljanov (1974).

### Saharo-Arabian region

According to Zohary (1973), the west of Iran is touched by the Saharo-Arabian region. Short and mild winters and long and dry hot summers are typical for this part of the country (Frey and Probst 1986). Two endemic planthoppers of Iran (*Callodictya kazeruna* (Dlabola, 1986) (Dictyopharidae) and *Phantia helleri* Linnavuori, 1962 (Flatidae)) have been recorded from this part while the former is in common with other parts of the Irano-Turan (Zagros Mt.) and the latter has a rather wide distribution in the Irano-Turanian and Nubo-Sindian regions (Fig. 2). Hence this region is not considered as an endemic zone in distribution patterns of endemic planthoppers.

### Irano-Turanian region

The region covers nine tenths of the land of Iran and major parts of the Iranian Plateau, from central to south of Asia. The region consists of plains, deserts and mountains which are divided into 8 primary divisions in this study: Kerman, Karkas Mt., Humid Zagros, Dry Zagros, North Khorasan Mt., South Khorasan Mt., Southern slopes of Alborz Mt. and Deserts and Plains.

#### Kerman Mountains

**Figure 3.**
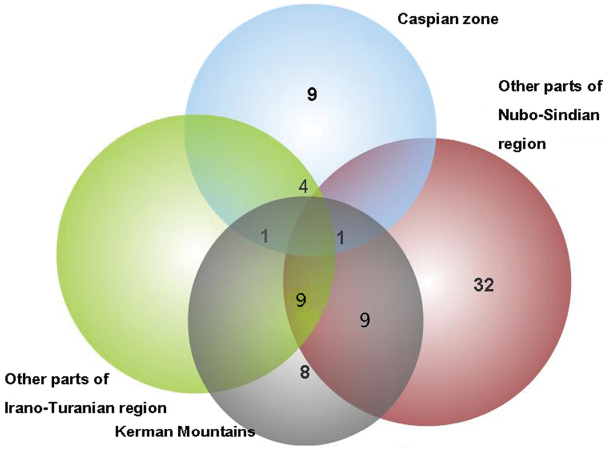
Venn diagrams for the number of endemic species in Kerman Mts and other areas with common species.

The complex of the Kerman Mts located in the southeast of the Irano-Turanian region and partially in the Nubo-Sindian (Fig. 1). The mountain range is a part of the Zagros chain but far from the other mountains and surrounded by deserts. The mountain and the deserts around have been considered as a zoogeographic zone by Zarudny (1911). Twenty eight endemic planthoppers of Iran were recorded from this mountain range, 8 of which endemic for there (Table 1, Fig. 3). The mountain range appears to be rather isolated from the Zagros and is limited by the Jazmurian lowlands in the south, Dasht-e Lut kavirs and other Irano-Turanian plains on the other sides. The isolation of mountains by plains and deserts creates a rather suitable condition for isolating populations and speciation. The high mountains in this complex (eg.: Hezar: 4,465 m, Lalehzar: 4,351 m, Tang-e Dalfard, 3,348 m) may be convenient places for relict species after the cold temperature of the Pleistocene ice age. However the fauna of endemic species is rather affected by both Nubo-Sindian (19 common species) and other parts of Irano-Turan (10 common species) (Fig. 3).

#### Zagros mountains (Humid and Dry Zagros and Karkas Mt.)

The Zagros mountain chain stretches from northwest of Iran to southeast near the Strait of Hormoz and is considered as the south branches of the Alpine-Himalayan orogenic belt (Dewey et al. 1986). The formation of the Zagros orogenic belt is subjected to controversy. Some geologists believe it has been made by the collision between the Afro-Arabian and Eurasian plate during the Cenozoic (Takin 1972, Agard 2005). As a result of this collision, several parallel folds were formed (Alan, 1969). The long mountain chain divides the Mediterranean climatic zone from the arid regions of west Asia. It is also a barrier between Mesopotamia and the Plateau of Iran with some corridors for the distribution of the fauna between the two zones (Anderson 1968).

In this study the Zagros chain was divided into 3 primary zones (Fig. 1): “Humid Zagros” in the northwest which consists of higher mountains and is affected by Mediterranean winds, “Dry Zagros” in the southeast with a drier climate and rather isolated mountains as the result of wider parallel valleys, and the Karkas mountain which appears to be a rather isolated mountain surrounded by Maranjab kavir and other lower lands around. Two different zones in northwest and southeast Zagros are also applied by Hedge and Wendelbo (1978) for distribution patterns of endemic phanerogamic plants, named: Armeno-Kurdic and Zagros, respectively. Similarly, Zarudny (1911) recognized the northwest of Zagros as a separate zone with a fauna similar to the Caucasus. Northwest of Zagros, (Armenian) was mentioned as a different area from southeast of Zagros, (Zagrossan) in Emeljanov (1974) as well. However both of them were considered as divisions of “Middle Eastern mixed”.

**Figure 4.**
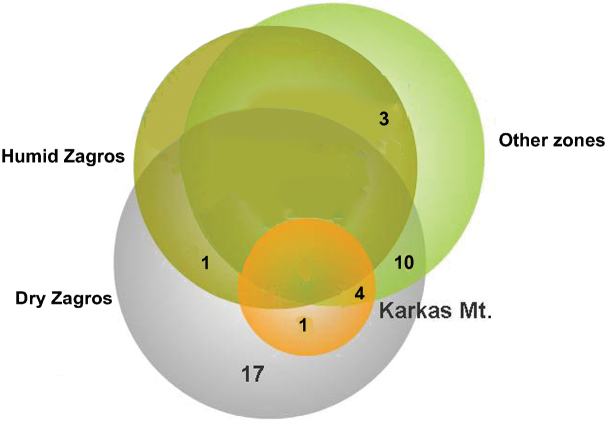
Venn diagram for the number of endemic species in Zagros Mts. (Humid Zagros, Dry Zagros and Karkas Mt.) and other areas with common species.

A total of 36 endemic planthoppers of Iran have been recorded from Zagros Mts, 19 of which are endemic to the Zagros (Table 1), 17 endemic to Dry Zagros, and 2 in common with Dry Zagros and Karkas Mt. or Humid Zagros (Fig. 4). The Venn diagram (Fig. 4) shows there are no endemic species for Humid Zagros and Karkas Mt. and the fauna of Iranian endemic planthoppers in those primary zones are mainly in common with Dry Zagros and partially with the south Alborz and Caspian zones. Therefore, those two primary divisions of the Zagros Mountains are not considered as endemic zones. Differently from the wall-like Alborz Mt., the Zagros chain comprises series of parallel ridges and valleys. The distances between mountains increases from northwest to southeast. Although the wider lower lands between the mountains in the south of Zagros (Dry Zagros) form corridors for the distribution and migration of species, mountains surrounded by those lower lands can provide a vicariant condition for speciation or at least prevent the distribution of species to other locations. The fauna of endemic planthoppers in Dry Zagros has some species in common with the Nubo-Sindian (10) and Kerman Mt. (2) which can be explained by the rather short distance between them.

#### Khorasan mountains

It is believed that Iran has been connected to central Asia since the Oligocene. Then the plateau of Iran was uplifted during the Tertiary orogeny and consequently Kope Dagh (Northeast of Iran), Hindukush and Himalaya were created (Anderson 1968). Mountains in northern Khorasan are a continuation of the Alborz but lack the wall-like integration of the Alborz and the humidity of the Caspian Sea, and mountains in both northern and southern Khorasan are rather parallel with the Zagros. In this study they were considered as different primary zones due to their distances and the distribution of endemic planthoppers.

**Figure 5.**
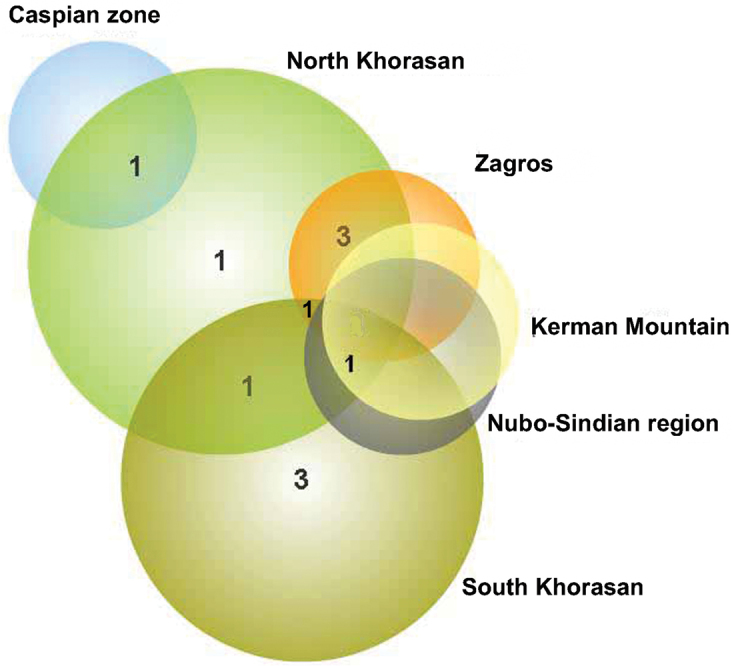
Venn diagram for the number of endemic species in Khorasan Mts and common species with other areas.

**Figure 6.**
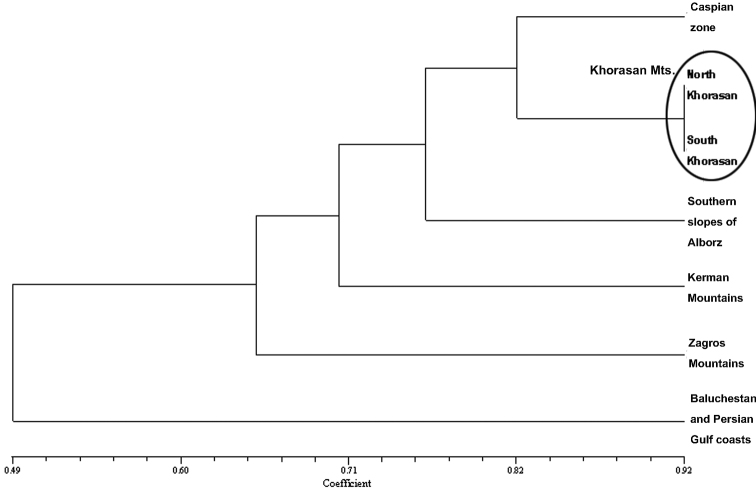
Cluster diagram for the endemic areas of Fulgoromorpha in Iran.

A total of 11 Iranian endemic planthoppers have been recorded from Khorasan Mts, 5 of which are endemic to this mountain range (Table 1), 3 in the south, 1 in the north and 1 in common (Fig. 5). The cluster analysis shows the similarity of the northern and southern fauna (Fig. 6). Hence the two mountains can be considered as a same zone in the distribution pattern. While south parts, which are surrounded by Kavir-e Lut and Dasht-e Kavir to the west and lowlands in Afghanistan to the east, have more endemic species and appear to be more suitable places for speciation.

#### Southern slopes of Alborz

The Alborz mountain chain represents a north branch of the Alpine-Himalayan orogenic system (Dewey et al. 1986). It stretches from northwest of Iran to southeast of the Caspian Sea and includes the highest mountain of west Asia, Damavand 5,670 m. It forms a consistent barrier between the fauna of the Caspian zone and the Plateau of Iran.

**Figure 7.**
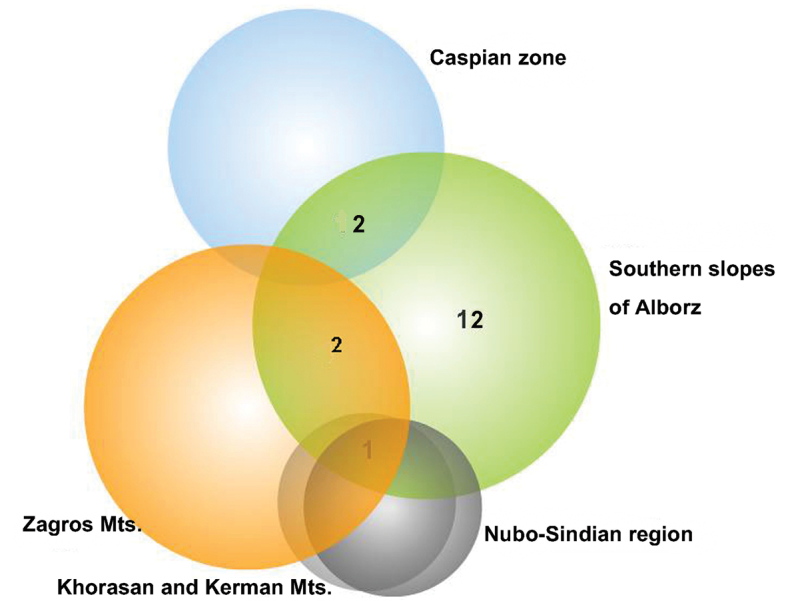
Venn diagram for the number of endemic species in southern slopes of Alborz and common species with other areas.

Seventeen endemic planthoppers of Iran are recorded from the south of Alborz, 12 of which endemic to the region (Table 1, Fig. 7). The southern slopes of Alborz have an absolutely different climatic condition from the northern Slopes due to the lack of corridors for receiving the humidity of the Caspian Sea. The presence of the Dasht-e Kavir desert in the south can be another factor for preventing the distribution of populations and probably speciation. There is only one common endemic planthopper between the southern slopes of Alborz and northern Khorasan whereas three species in common with the Zagros show a relationship with the western mountains.

#### Deserts and plains

Two big salt pans or Kavirs in central Iran are undrained basins and the lands surrounding them have typical features such as clayey or sandy soil and a high amount of salt on the surface. The inner lands of these salt marshes are nearly free of vegetation (Hangay and Szekely 2005, Frey and Probst 1986, Zohary 1973). It is said that Dasht-e Lut is considered as the hottest place in the world exceeding 70°C of temperature in some parts of it (Mildrexler et al. 2011). There are marshes and mud grounds with large crusts of salt in inner parts of Kavirs while some parts have a steppe-like appearance and some other regions are surrounded by *Tamarix* spp., *Calligonum* spp. etc. (Frey and Probst 1986). Hedge and Wendelbo (1978) and Anderson (1968) recognized deserts and plains of the central Plateau of Iran as biogeographic zones. However Zarudny (1911) did not mention them in his list. The distribution patterns of the Iranian endemic planthoppers appear to be very restricted in central Iran. *Malenia masirica* Dlabola, 1986, *Mesophantia pallens* Melichar, 1902 and *Persepolia servadeina* Dlabola, 1982 are the only endemic species recorded from this part of the country while none of them are endemic for those deserts. The two first species have a rather wide distribution in Iran and the third one has been collected so close to Kerman Mt. Few records of endemic Iranian planthoppers from the deserts may be simply because of less collecting in those dry areas or poor vegetation in some parts of them. None the less, according to our current knowledge, deserts and plains of the Irano-Turan cannot be considered as an endemic zone for planthoppers.

### Nubo-Sindian region

This part of the country in southern and southeastern Iran is the only region which is not considered as Palaearctic but as a part of the Oriental. A varied terminology has been used for this region. Hedge and Wendelbo (1978) used the term “Sahara-Sindian” to show a west to east continuum from southwest Asia to Send deserts while Frey and Probst (1986) preferred Nubo-Sindian because they believed the distribution patterns of the Nubo-Sindian province are obvious here while Saharo-Arabian and Mediterranean species have migrated to this region.

**Figure 8.**
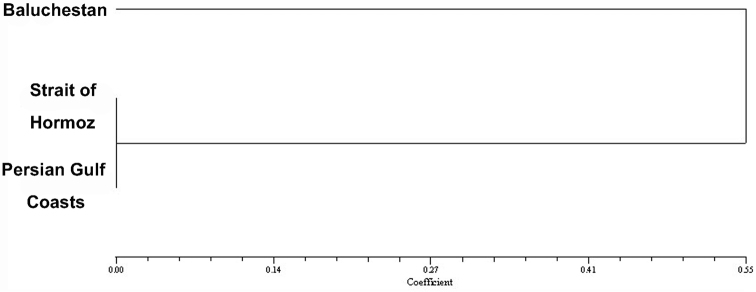
Cluster diagram for the primary zones of endemic Fulgoromorpha in tjr Nubo- Sindian region.

**Figure 9.**
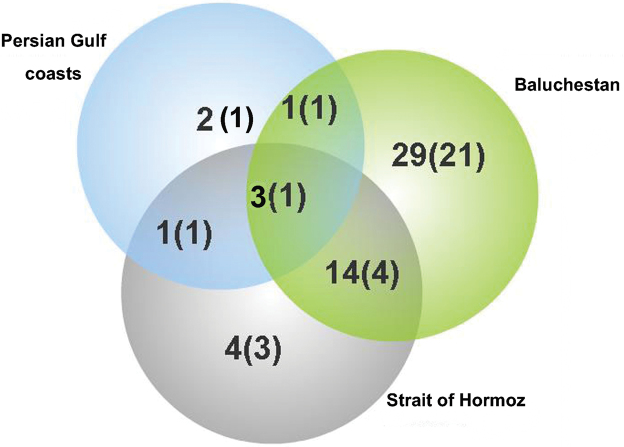
Venn diagram for the number of endemic species of the Nubo-Sindian region in three primary zones (Numbers in the parentheses are the number of endemic species in that specific primary zone)

By subtracting the endemic species of a small part of the Kerman Mountain from the Nubo-Sindian, 54 recorded endemic species of Iran, which is more than half of the total, are distributed in this region and 32 of these are endemic to the region (Table 1). The distribution of endemic Fulgoromorpha shows three rather separate groups which are recognized as three primary zones here: Baluchestan, the Strait of Hormoz and the coasts of the Persian Gulf (Fig. 1). The cluster analysis showed the similarity of the fauna of the endemic species in the Strait of Hormoz and the Persian Gulf coasts (Fig. 8) so those two primary zones can be considered as a same zone. Anderson (1968) found a discontinuity in the faunal elements of southern and southeastern Iran and justified it with the relatively recent position of the Persian Gulf and lower sea levels during the Pleistocene which must have caused dry land contact between Iran and Arabia. This belief is confirmed by evidence for ecological continuity across the two sides of the Persian Gulf. Therefore the higher sea levels after the ice age can also produce a vicariant condition in the Persian Gulf coasts and isolating endemic species between mountains and a different climate condition to the north and the waters of the Persian Gulf to the south. The number of endemic species in the Nubo-Sindian is reduced from east to west which may be because of the isolation of the eastern species by the Kavie-e Lut and Jazmurian lowlands. On the other hand, the Venn diagram (Fig. 9) shows a rather high number of common species between Baluchestan and the two other adjacent areas. Hence according to our current knowledge of distribution patterns of endemic planthoppers, the Baluchestan primary zone can also be united with the other primary zones of the Nubo-Sindian region. The area was also considered mainly as one division (Makran mixed) with a very small area of Baluchestan in southeast (Sind plane) by Emeljanov (1974). According to Ghorbani (2002), some parts of Baluchestan, the Strait of Hormoz and the area between them had been occupied by the old Tethys so the common endemic species between Baluchestan and two other primary zones in southern Iran should have crossed the area after drying.

**Figure 10.**
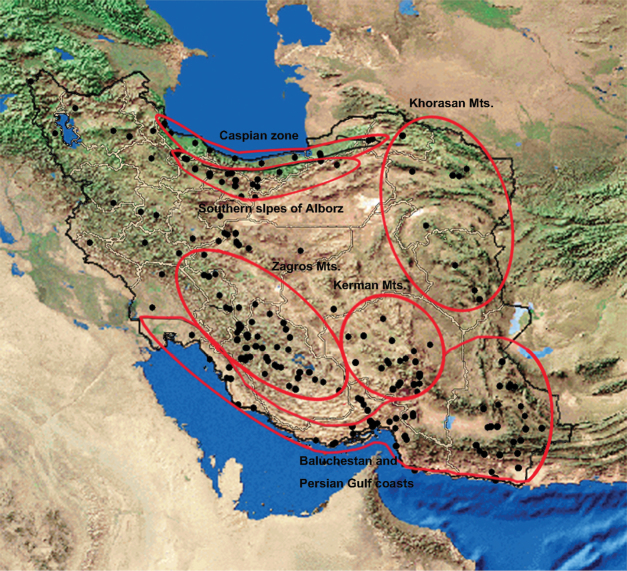
Final endemic zones and distribution of endemic Fulgoromorpha of Iran.

**Figure 11.**
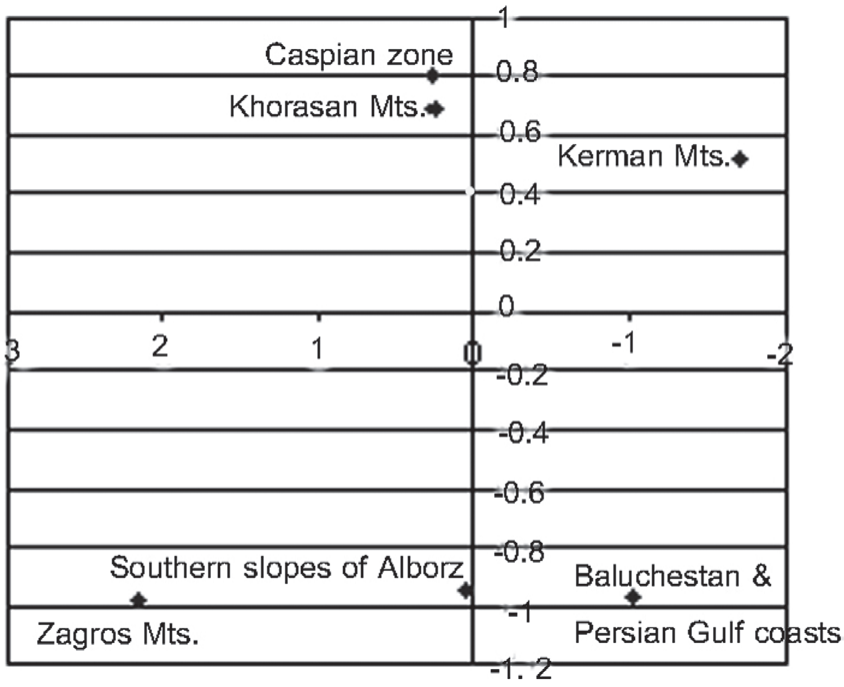
A similarity map of endemic zones of Fulgoromorpha of Iran resulted from Aslcal analysis.

The final 6 endemic zones (Caspian zone, South slopes of Alborz, Khorasan Mts, Zagros Mts, Kerman Mts and Baluchestan and Persian Gulf coasts) are shown in Fig. 10. The cluster analysis on six final endemic zones of Iranian planthoppers (Fig. 6) indicates Baluchestan and Perisan Gulf coasts the only region which is considered as a part of the Oriental Region, as the most different zone from the others. Then Zagros Mts, Kerman Mts, Alborz, Khorasan Mts and Caspian zones are diverging, respectively. Alscal analysis produced a map according to the similarity of endemic zones. Rotating the similarity map canvas horizontally (Fig. 11), makes it more comparable with the geographic map (Fig. 10). The Caspian zone and the Nubo-Sindian region are located in similar situations in both maps. While the Khorasan zone is closer to the Caspian zone in the similarity map rather than its geographic situation. This similarity can be justified by the possibility of specimens migrating between two zones at the end of the wall-like mountains of Alborz at the east of the Caspian zone. This is why the southern slopes of Alborz go farther in the Alscal map and confirms the efficiency of the barrier of the Alborz mountain in separating species of the two adjacent zones. Although the Zagros mountain has rather similar situations in both maps, it is farther from the Nubo-Sindian in similarity rather than its geographic situation. The reason may be the higher number of endemic species in Baluchestan rather than in the coasts of Persian Gulf and the Strait of Hormoz close to the Zagros Mountains. The situation of the Kermann Mts in the similarity map moved from its geographic situation towards the north. The rather far distance of this zone from the others, confirms the isolation of the species living there. However, deleting the doubtful record of *Morsina persica*, common between the Caspian zone and the Nubo-Sindian part of the Kerman Mountains may move the situation of the Kerman zone in the similarity map towards the south.

The records of the endemic planthoppers of Iran belong to recent 110 years (Mozaffarian and Wilson 2011). The nature of Iran, like any other land, has been exposed to various changes during this long period by events, such as agriculture, war, fire in woods and forests, overgrazing, urbanization and so on. Hence, probably the presence of some endemic species with a limited distribution and the probable extinction of some of them should be subjected to further investigations.
